# Modelling the participation decision and duration of sporting activity in Scotland

**DOI:** 10.1016/j.econmod.2009.10.003

**Published:** 2010-07

**Authors:** Barbara Eberth, Murray D. Smith

**Affiliations:** a Health Economics Research Unit, University of Aberdeen, Foresterhill AB25 2ZD, Scotland, UK; b Health Economics Research Unit, University of Aberdeen, Foresterhill AB25 2ZD, Scotland, UK

**Keywords:** Sport, Sample selection, Participation, Duration, Copula

## Abstract

Motivating individuals to actively engage in physical activity due to its beneficial health effects has been an integral part of Scotland's health policy agenda. The current Scottish guidelines recommend individuals participate in physical activity of moderate vigour for 30 min at least five times per week. For an individual contemplating the recommendation, decisions have to be made in regard of participation, intensity, duration and multiplicity. For the policy maker, understanding the determinants of each decision will assist in designing an intervention to effect the recommended policy. With secondary data sourced from the 2003 Scottish Health Survey (SHeS) we statistically model the combined decisions process, employing a copula approach to model specification. In taking this approach the model flexibly accounts for any statistical associations that may exist between the component decisions. Thus, we model the endogenous relationship between the decision of individuals to participate in sporting activities and, amongst those who participate, the duration of time spent undertaking their chosen activities. The main focus is to establish whether dependence exists between the two random variables assuming the vigour with which sporting activity is performed to be independent of the participation and duration decision. We allow for a variety of controls including demographic factors such as age and gender, economic factors such as income and educational attainment, lifestyle factors such as smoking, alcohol consumption, healthy eating and medical history. We use the model to compare the effect of interventions designed to increase the vigour with which individuals undertake their sport, relating it to obesity as a health outcome.

## 1 Introduction

Physical activity and fitness contribute positively to the health, well being, and quality of life of all individuals regardless of their age. Despite the health benefits associated with physical activity, unhealthy lifestyles characterised by physical inactivity, over-consumption of tobacco and alcohol, and unhealthy diets are major risk factors for premature death and chronic diseases such as coronary heart disease, type 2 diabetes, hypertension and various types of cancer. The correlation between unhealthy lifestyle behaviours and chronic diseases has been of great policy concern (World Health Organisation, 2005) given that the adverse effects of unhealthy lifestyle choices can be prevented through behavioural changes. Regardless of the well-known health benefits resulting from a physically active lifestyle, World Health Organisation Europe (2007) report that at least two thirds of the adult population of the EU countries are insufficiently physically active for optimal health benefit. For the Scottish population only 41% of men and 30% of women achieved the recommended physical activity guidelines in 1998 which increased slightly to 44% of men and 33% of women aged 16–74 in 2003. These figures encompass physical activities during home, work and leisure time in addition to daily walking activities (Scottish Health Survey, 2003). Physical inactivity has further been identified as one of the important risk factors associated with weight gain and, consequently, obesity; the latter becoming a topic of increasing health policy concern on the backdrop of the alarming increase in obesity prevalence witnessed worldwide. Unhealthy lifestyles in general and their detrimental effect on mortality were the focus of the World Health Organisation report *Preventing chronic disease: A vital investment* (World Health Organisation, 2005), estimating that each year at least 1.9 million die of diseases induced by physical inactivity. Not surprisingly, promoting physical activity is one of the top priority areas identified by the World Health Organization (2002) and the European Association for the Study of Obesity (World Health Organisation Europe, 2007), highlighting the urgent need for understanding the influences that motivate individuals to undertake physical activity, and equally those influences that diminish activity.

Physical activity is most usefully expressed as a function of the intensity with which it is carried out, how often and for how long it is undertaken. Epidemiologic research defines physical activity as any bodily movement produced by skeletal muscles that results in energy expenditure (Caspersen et al., 1985). This definition encompasses all types of movements and can be classified according to type and intensity. The simplest categorisation in terms of type relates to an individual's daily activities which can be segmented into occupational, transportation, household and leisure time activities. A further sub-categorisation can be applied to leisure time activity such as household (DIY, gardening, cleaning) and sports activities. The intensity with which these physical activities are performed can be usefully expressed to be of low, moderate and high intensity, or inactivity. Defining physical activity type and intensity as such allows for meaningful measurement. In epidemiological studies intensity is often measured in terms of metabolic equivalent tasks (METs) estimating the rate of energy expenditure; see Ainsworth et al. (2000) for a compendium of MET values for various types of physical activities. However, epidemiologists do acknowledge that physical activity presents measurement challenges, as evidenced by the different approaches proposed in that literature; see Hu (2008) for a summary of these. Objective measures of physical activity measurement in terms of total energy expenditure are the method of doubly-labelled-water (DLW) and indirect calorimetry, while direct measures of physical activity include the use of pedometers, accelerometers and heart rate monitors. Both sets of measures have their advantages and disadvantages. DLW and indirect calorimetry impose participation burden and are costly to implement. They further cannot distinguish between different types of physical activity. The second set of measures also impart a financial cost and may not be feasible to use in large population studies. Large epidemiological studies therefore most commonly employ physical activity questionnaires due to their practicality, low cost implications and low burden on participants. These questionnaires gather self-reported accounts of physical activity behaviours. They typically collect information on the types of physical activity undertaken, frequency, duration and intensity (Welk et al., 2005). However, it should be noted that one of the potential disadvantages of using self-reported information of physical activity behaviours is the tendency for an individual to overstate their dimensions of physical activity and to understate their sedentary behaviours. The physical activity information in the data used here is self-reported, but it does have the advantage that it provides comprehensive information on respondent physical activity type, intensity, frequency and duration.

The importance of the duration, frequency and intensity of physical activity behaviours can readily be seen in policy prescriptions used to promote physical activity and fitness. For example, the Scottish government in their 2003 report *Improving Health in Scotland — The Challenge* (Scottish Executive, 2003) recommend adults undertake 30 min of moderate physical activity on at least 5 days per week in order to maintain a healthy weight. The decision of individuals over whether or not to participate in physical activity is a further factor that must enter into consideration. The Scottish recommendation aims to increase the numbers of physically active adults to 50% of the population by 2022.

Understanding why many individuals do not meet the recommended physical activity guidelines may derive from a lack of evidence in terms of the effect of economic and demographic factors that determine sports participation. Economics lends itself well to answer this question since it offers theoretical models about how individuals make choices regarding the allocation of their time to different activities and how these are influenced by their economic circumstances, environmental influences and demographic characteristics. The idea was originally formalised in the income–leisure trade-off model of labour supply (Becker, 1965). In Becker's model, the unit of analysis is the household. Individuals within a household derive utility from the consumption and production of ‘basic' commodities such as a visit to the cinema, or having dinner together, by combining time and market goods. In terms of the income leisure trade-off, the production and consumption of basic commodities requires time which is time not spent at work. An example of one such commodity is sporting participation. Drawing on Becker's work, Cawley (2004) uses this framework to derive the so-called SLOTH model of time allocation that incorporates the idea that individuals produce their own health. The underlying assumption of the SLOTH model derives from the observation that individuals choose how to allocate their available time across activities such as sleeping, leisure, work, transportation and home production in order to maximise utility given financial, time and biological constraints. Humphreys and Ruseski (2006) (HR hereafter) extend the SLOTH model further to allow for recreational demand in order to integrate and analyse decisions of physical activity consumption and their durations, enabling evaluation of how economic factors such as income and education as well as time considerations impact on sports participation and duration. The importance of a lack of time to participate in sports has recently been highlighted in the 2006 report *Sport, exercise and physical activity: public participation, barriers and attitudes* (Scottish Executive Education Department, 2006) in which a lack of time is found to be one of the most cited reasons for physical inactivity next to a lack of accessibility and availability of facilities and health considerations, those results were based on data sourced from the Scottish Social Policy Monitor.

The analysis presented here offers an evaluation of the appropriateness of the Scottish physical activity recommendation in achieving its desired effect. We will examine the extent to which participation and duration of physical activity are associated by testing whether these variables can be studied independently of one another. Furthermore, our modelling approach will provide robust identification of the economic, demographic, health related and lifestyle determinants of the decision to engage in physical activity and the duration thereof. We will study the Scottish policy in terms of conditional analyses designed to show how the model results can be used to predict changes in health outcomes such as BMI. For example, we use our fitted model to predict duration changes resulting from an increase in vigour from a low degree of effort to a moderate degree of effort. The resulting change can be used as input in a health outcome context — we choose obesity — to infer if the resulting change in the attributes of physical activity have significant downstream health effects.

The environments within which opportunities arise to engage in physical activity can be split into three spheres: the home, the workplace, and during leisure time. The economic literature argues that there exist environmental factors that serve to discourage participation in physical activity given technological advances in home and workplace, leading to an increase in sedentary behaviours. With regards to the workplace, Lakdawalla et al. (2005) and Philipson and Posner (2004) argue that the shift from strenuous manual to less strenuous non-manual work increases the cost of physical activity during leisure time. Other themes explored in understanding the environmental obstacles to participation include trends in television viewing, the increased use of automobiles, and the effect of infrastructure relating to the availability of recreation, sports and health facilities; see, for example, French et al. (2001), Brownson et al. (2001, 2005), Ewing et al. (2003), Sturm (2004) and Hill et al. (2003). Irrespective of these considerations, the evidence base relating to the individual determinants of physical activity behaviours is scarce, possibly reflecting a lack of data availability. Evidence relating to the effectiveness of physical activity intervention is also thinly spread, one exception though is Hillsdon et al. (2004).

Farrell and Shields (2002) (FS hereafter) investigated the economic and demographic determinants of participation for adults for ten sporting activities using data sourced from the 1997 Health Survey for England. Their two main policy conclusions were that income is an important factor in sports participation in England, lending support to policies that aim to make sporting facilities financially accessible across all income groups in society. They further argue that increased sports participation is a promoting factor for social inclusion and health improvement for socially disadvantaged members of society. HR and Downward and Riordan (2007) (DR hereafter) extend the analysis to incorporate decisions on sports duration. The former use data on adults from the 2000 Behavioural Risk Factor Surveillance System, while the latter employ data sourced on adults from the 2002 General Household Survey. Whilst HR focus on the economic determinants of participation in physical activity and sports, DR change tack and focus on the role of investment in social capital and social interactions as a determinant of sports participation and frequency thereof. It is argued in both articles that sports participation should not be viewed in isolation from the duration decision. Ignoring this type of selectivity can introduce unwanted statistical biases into model estimates, that in turn can fuel adverse consequences for policy prescriptions. Returning to HR, they find a similar positive effect of income on participation to that of FS. Whilst the effect of income on participation is positive, HR also show that higher income reduces time spent in sporting activities conditional on participation, a result mirrored in the analysis of DR. This supports the notion that the opportunity cost of time is an important element of both the participation and the duration decision, and one which needs to be addressed in any policy recommendation. All three articles — HR, FS, DR — stress the importance of household characteristics such as the presence of children on sports participation and duration, as well as the effects of age, gender, and marital status. Males have consistently found to be more likely to participate in sports relative to women, that sports participation is decreasing in age, and that married individuals are less likely to participate in sports relative to non-married individuals. Lifestyle factors have also been found to be significant determinants of sports participation and duration. Both are increasing in subjective health measures and are positively associated with alcohol consumption but negatively related to smoking. Our modelling approach relates to this literature in that we will also incorporate these types of determinants. However, we also introduce additional factors that have previously not been investigated. Front and foremost these relate to the vigour with which sports are undertaken, which we believe to be an important factor in considerations of duration. Whilst HR and DR present their analyses for various types of sporting activity, we do not make such distinction in the present paper because we embed our analysis of sports participation and duration into the current Scottish policy recommendation, which applies to the participation, duration and intensity of sports in aggregate. However, we do present the model results by gender. We also take our analysis a step further in that we investigate the effect of the Scottish policy, relating the results from our model to predict changes in obesity.

Whilst we can think of endless types of physical activities carried out during home, leisure and work time, the main focus here is on sporting activities undertaken during leisure time, we exclude any physical activities undertaken during home and work time. Physical activity relating to day-to-day walking activities are also excluded from our construction of sporting activity.

The paper proceeds in Section 2 to describe the data and construction of the key attributes of sporting activity. Then, in Section 3, the econometric model is set within the context of a sample selection model. Empirical results are presented in Section 4, including conditional analyses. Some conclusions are offered in Section 5.

## 2 Data

### 2.1 Scottish Health Survey

Data for this study are gathered from the 2003 Scottish Health Survey (SHeS), in which individuals self-report a wealth of health information (some of which is independently nurse-measured) as well as a large range of personal demographic and economic data. Our estimation sample comprises all adults (apart from pregnant women) aged between 16 and 64 years who also had a BMI between the values of 20 and 40. This gave us a sample of *n* = 4380 individuals. Of this number *n*_1_ = 2327 report to engage in sporting activities, corresponding to a sample participation rate of 53.1%.

### 2.2 Vigour, duration and multiplicity

The main data preparation task involves summarising individual sporting activity in terms of three basic components corresponding to the Scottish policy recommendations: the total time of involvement *T*, the number of events undertaken *Q*, and the degree of vigour at which sporting activities are undertaken *V*.^1^

In the SHeS, respondents report counts and averages calculated on sporting activities undertaken across the 28 day period prior to interview.^2^ In particular, reported are: (i) the number of days in the past 28 when each of a range of *J* types of sport were played^3^ (denote this by *d*_*j*_, *j* = 1,…,*J*), (ii) the 28-day aggregate duration of time spent playing sport *j* averaged by *d*_*j*_ (denote this by *a*_*j*_, *j* = 1,…,*J*), and (iii) whether the effort exerted on each sport (denote this by *e*_*j*_) was usually enough to make the respondent out-of-breath or sweaty (*e*_*j*_ = 1) or neither (*e*_*j*_ = 0).

Focusing first of all on vigour, we combine the individual response *e*_*j*_ with a non-individualised intensity classification *s*_*j*_ that is assigned to sport *j*. The latter was developed in the 1995 Scottish Health Survey (Scottish Office Department of Health, 1997); *s*_*j*_ = 1,2,3 classifies, respectively, sport *j* as being of low, moderate, high intensity. The 4-level combined classification

$${\tilde{\upsilon }}_{j}=s_{j}+e_{j}$$

represents an individualised categorical measure of vigour: low (*υ̃*_*j*_ = 1) fair (*υ̃*_*j*_ = 2) moderate (*υ̃*_*j*_ = 3) high (*υ̃*_*j*_ = 4). Once constructed, count numbers were such that it was necessary to combine low and fair into one class to yield observations *υ* = 1, 2, 3 on vigour *V*, where low vigour *υ* = 1 if *υ̃* = 1 or 2, moderate vigour *υ* = 2 if *υ̃* = 3, and high vigour υ = 3 if *υ̃* = 4. For example, if an individual reports exerting little to no effort (*e* = 0) on moderate-intensity swimming (*s* = 2) then for that sport they are assigned a low degree of vigour *υ* = 1 as *υ̃* = 2 Amongst participators, 12.3% are classified as undertaking sport with a low degree of vigour, 25.4% with moderate vigour, and 62.3% with a high degree of vigour.

Next, we define the total time of involvement in sporting activities over the 28-day period of recall. Duration *T* is observed with value *t* > 0 for a given individual according to the scheme:

(1) $$t=\overset{J}{\underset{j=1}{\sum }}a_{j}d_{j}1\{\upsilon_{j}=\max(\upsilon_{1},...,\;\upsilon_{J})\}$$

where the binary indicator 1{*A*} = 1 if event *A* is true, 0 otherwise. The purpose of the indicator appearing in (1) is to include into aggregate duration only those sports undertaken at the maximal degree of vigour observed for that individual.

Multiplicity concerns the number of events an individual undertakes. Because the data record limited information on any one event then the best we can say is that aggregate duration (1) results from the individual undertaking a multiplicity count of *Q* events observed with value *q* according to:

$$q=\overset{J}{\underset{j=1}{\sum }}d_{j}1\{\upsilon_{j}=\max(\upsilon_{1},...,\;\upsilon_{J})\}\text{.}$$

Implicit in this formula is the assumption that only one event can occur per day on any given sport. There is however little alternative open to us to alter this assumption because *d* is the only multiplicity variable recorded in the SHeS.

**Fig. 1:**
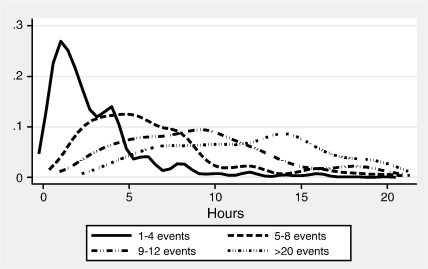
Gaussian kernel smooth duration distributions by grouped event multiplicity.

Fig. 1 shows kernel smooth aggregate duration distributions in units of hours per 28 days, where individuals have been grouped according to increasing multiplicity of events (those depicted are grouped as 1–4, 5–8, 9–12, and 12+ events per 28 days). The distributions shift progressively to the right as the multiplicity increases, implying that more time is devoted to sports as the frequency of events rises. Note also that the duration distributions become more spread with increasing number of sporting events. For instance, the average aggregate duration for 1–4 events is 3 h with a standard deviation of just over 3 h. These statistics more than double to just under 8 h with a standard deviation of 7.25 h for the next group that report 5–8 events per 28 days. Finally, for respondents reporting more than 20 events per 28 days the average aggregate duration is 29 h with a standard deviation of 22 h.

**Fig. 2:**
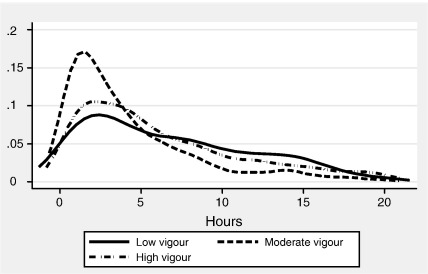
Gaussian kernel smooth duration distributions by vigour.

Fig. 2 depicts the aggregate duration distributions according to level of vigour: low, moderate, high. Note that all three distributions are roughly shaped as Gamma distributed variables. The distributions clearly show that high vigour individuals are more concentrated on lower durations as compared to individuals who exercise with moderate or low vigour. This is what we would expect to observe given that burn out will set in sooner for high vigour individuals compared to moderate and low vigour individuals. Nevertheless, the spread of all three vigour duration distributions is similar. Mean duration for the low and high vigour groups are slightly closer to one another compared to the average sport duration for the moderate group.

Table 1 presents counts of individuals undertaking sporting events (grouped into increasing multiplicity 1–4, 5–8, 9–12, 13–16, 17–20, 20+) by degree of vigour, where again it is events per 28 days. In general, we observe the majority of individuals who participate in sports undertake relatively few events irrespective of the degree of vigour, with 1031 out of the total of *n*_1_ = 2327 undertaking between only 1 and 4 events per 28 days. Indeed, for those whose sporting activities are rated at low and moderate vigour just over 55% undertake between 1 and 4 events per 28 days. This rate drops to around 37% for high vigour individuals, implying that this group tend to play sport on more occasions; their average is a little over nine events per 28 days.

### 2.3 Other covariates

For men the average time spent per week undertaking sports is 2 h and 25 min and for women it is 1 h and 28 min, a difference of about an hour per week. 54% of the overall sample (including those not actively engaging in sports) are women and 46% are men, note that the gender dummy is Male = 1. Amongst participants, 48.6% are men, whilst amongst the non-participants the share of men is slightly lower. The average age in the sample is 42.5 years. The average participant is 40 years old whilst the average non-participant is 46 years old. We categorised age into 10-year bands: 16–24, 25–34, 35–44, 45–54 and 55–64, the latter acting as the reference group. Participants are represented across all age groups, and in particular from ages 25 to 54. Only a small proportion of non-participants are aged 16–34, whilst the majority are aged 45–64.

Marital status is categorised into binary variables where being married serves as the reference group, with the other groups being married or cohabiting, and divorced, widowed, or separated. 65.4% of participants are married or cohabiting whilst the share is slightly higher amongst non-participants. Only 10.4% of participants are divorced, widowed or separated compared to 14.1% in the non-participant group. Other demographic variables include the number of children in the household aged 2–15, the number of infants in the household who are under 2 years of age, and a binary educational variable indicating whether the individual does not have an educational qualification, where holding an educational qualification is the reference group. Interestingly, 63% of participants have children aged 2–15 compared to 47% of the non-participants. Having children might be seen as a barrier to participate in sports but the figures presented here clearly suggest otherwise. We elected to use the indicator ‘natural mother still alive’ as proxy for available child care (even though in the SHeS it is not known if the mother lives in the vicinity of the son/daughter).

The set of variables relating to the respondent's health include self-reported general health, psychological well-being, and presence of a limiting long-standing illness. Self-reported general health is coded into four binary variables: very good, good, fair, and bad or very bad general health. The very bad general health dummy variable serves as the reference group. 86% of participants report very good or good general health compared to 61% of non-participants who have a higher share reporting fair and bad general health. Psychological well-being is coded into four binary variables: good well-being, bad well-being, fair well-being, and observation missing; the reference group is bad well-being. Presence of a limiting long-standing illness is coded into it being present, being present but non-limiting and altogether absent; the latter we chose as the reference group. Whilst both participants and non-participants report similar figures for absence of a limiting long-standing illness, participants report considerably less of a presence of a limiting long-standing illness, and both groups report similar presence of a non-limiting long-standing illness. As a final health variable we elected to use a binary variable indicating whether the respondent had an accident in the past 12 months. Interestingly, more participants compared to non-participants report having had an accident in the last 12 months.

The economic variable employment status was categorised into four dummy variables: employed, unemployed, retired, and economically inactive. Employment is taken as the reference group. The majority of respondents in both groups are employed, this share is higher amongst participants where we also find a slightly higher share of unemployed, but a considerably smaller share of the economically inactive compared to non-participants. A further economic variable is the natural logarithm of equivalised household income.

Lifestyle behaviours are summarised by alcohol consumption patterns, smoking status, time spent watching television, a summary measure of diet and area level indicators for average physical activity duration and BMI levels in the health board area the respondent lives in. Smoking is categorised into current smokers and ex-smokers with reference group non-smokers. 50.7% of participants report never to have smoked compared to 39% of non-participants. Whilst 23.3% of participants are smokers, 35.5% of non-participants indicate to be smokers. The percentage of ex-smokers in both groups is similar. Alcohol drinkers are separated into those indulging in regular alcohol consumption above the official weekly guideline limit, and those who consumed less than the official weekly guideline limit. The reference groups are individuals who never or occasionally consume alcohol. Interestingly, 41% of non-participants only drink occasionally or have never done so. This is in stark contrast to participants for which only 29% indicate that they are occasional drinker or don't consume alcohol at all. 45.4% of participants and 38.7% of non-participants regularly drink alcohol under the limit. On the other hand, 19.7% of non-participants regularly drink alcohol over the limit compared to 24.2% of participants. The number of hours spent watching television per week is measured as a continuous variable. Participants watch on average 2 h less television per week than non-participants. A healthy eating score variable was constructed using a scoring system based on the selection of five healthy foods (fish, poultry, potatoes, fruits and vegetables) and five non-healthy foods (chips, crisps, confectionery, biscuits and soft drinks). Respondents are scored points on the basis of the frequency that they consumed both healthy and non-healthy foods with a score of zero pertaining to most unhealthy and a score of three pertaining to healthiest. Individual scores for all food types consumed were then summed up to a final score ranging from 0 (most unhealthy) to 30 (most healthy). The healthy diet score is on average one point higher for participants than it is for non-participants.

We construct an area measure of sport activity measuring the average number of hours of sports per week in each Health Board. The relationship between the duration of sporting activities at the individual and the Health Board area level can be thought of as a peer group effect. It is a measure of physical activity level in the area population and summarises the contributing environmental factors impacting on sport activity behaviours at the individual level. These we interpret to include factors such as the availability of sports facilities, attitudes towards sport, diet behaviour and deprivation held generally across the area in which the respondent lives, all of which should correlate with individual time participating in sport activities. Further, average area sport activity level, holding all other characteristics of the ‘local’ population constant, should also affect individual sport activity since the former is an indicator of social norms.^4^ The average of the average weekly number of hours of sporting activity across Health Boards is 1.87 amongst participants and 1.82 amongst non-participants. The average BMI in each Health Board can be interpreted similarly in terms of peer group effects. The overall average is 27 which is in the overweight range.

## 3 Econometric model

### 3.1 Introduction

In this section we set out our econometric model that takes into account the selection issues relating to the decisions to participate in sporting activities and the duration with which sporting activity is undertaken. Selectivity is frequently a problem with microeconometric data whereby underlying individual circumstances can themselves influence the observations collected on random variables. Statistical models of increasing complexity have been constructed to account for selectivity in its various guises, should it be present, with the classic example in economics being labour force participation and wage offers, where the distribution of wages is truncated by unobserved reservation wages; see Gronau (1974) and Heckman (1974). The same conceptual framework applies in our setting because we examine the propensity to participate and, contingent upon participation, the factors affecting duration lengths. If there exists an endogenous relationship between the variables then sample selection biases enter if, for example, duration is modelled independently of participation. We test for whether association is present or not in the context of binary models designed to allow for possible data selectivity (Table 2).

Both HR and DR in their investigations of the determinants of participation and duration decisions of sporting activities adopt the self-selection framework. We however use the ‘copula approach’ to model specification as it allows us to treat correctly the distribution of the duration variable as supported on the positive part of the real line. The distributional specifications underpinning the models examined by HR and DR err by imposing normally distributed durations.

The copula approach is a modelling strategy derived from the representation theorem due to Sklar (1959, 1973) whereby a joint distribution is induced by specifying marginal distributions and a copula function, where the latter binds together the margins to form the joint distribution. The copula parameterises the dependence structure of the random variables. This then frees the location and scale structures to be parameterised through the margins, one at a time. Most importantly, the copula approach permits specifications other than multivariate Normality, although it does retain that distribution as a special case. Nelsen (2006) surveys copula theory.

In our self-selection model a binary indicator *S* governs whether or not an observation is generated on a duration random variable *T*. Selectivity arises if *S* and *T* are correlated, or associated. Importantly, of concern is whether sports participation can be studied independently of sporting duration lengths. A priori it is difficult to predict whether there will be a positive or negative association between participation and duration. For example, we might expect either type of association between participation and duration if individuals in the labour force have to make work/leisure trade-offs. Employees may only have limited opportunity to engage in sports during leisure time due to their prescribed time constraints. Once the decision to participate has been made, we may observe the individual to engage in physical activity of shorter duration, a negative association. On the other hand, individuals who are in work may be more aware of the need to engage in sports to achieve a healthy work–life balance and will therefore be observed to engage in longer durations. They value added benefits such as the ability to concentrate for longer time periods at work and feeling better about themselves, hence a positive association.

### 3.2 Observation rules

Following the general copula modelling procedure described in Smith (2003), we embed the self-selection model within a latent utilitarian framework that can be transformed to observed variables as described by a set of observation rules. The first utility is the propensity to participate in sporting activities. Denoted by *S*⁎ this is a latent, continuous random variable defined throughout the entire real line. We relate it to the observable participation variable *S* as per

(2) $$S=1\{S^{⁎}>0\}$$

where the binary indicator 1{*A*} = 1 if event *A* is true, 0 otherwise.

The second utility is the propensity of time spent undertaking sporting activity. This is latent and continuous, and defined on the positive part of the real line. We denote it by *T*⁎ and relate it to the observable duration variable *T* by

(3) $$T=1\{S^{⁎}>0\}T^{⁎}$$

implying that the propensity coincides with the observed duration only amongst those observed to participate. Together the observation rules (2) and (3) describe the relationship between the utilitarian variables (*S*⁎,*T*⁎) and the observed variables (*S*,*T*).

### 3.3 Modelling assumptions

Modelling assumptions we impose begin with a Normality assumption for participation propensity; i.e. *S*⁎ ~ *N*(*x*′*β*,1) so that

(4) F(s⁎)=Pr(S⁎≤s⁎)=Φ(s⁎−x′β)

where *s*⁎ is real-valued, regressors *x* (*k* × 1) parameter *β* (*k* × 1) and Φ(·) denotes the cumulative distribution function (cdf) of the standard Normal distribution. A unit variance is imposed for identification purposes because all scale information on *S*⁎ is lost in the transformation (2) to the observed variable *S*. Clearly, given (2) and (4),

$$\mathrm{Pr}(S=s)={\left(1-\Phi \left(x^{\prime }\beta \right)\right)}^{1-s}\Phi {\left(x^{\prime }\beta \right)}^{s}$$

for *s* = 0,1.

We assume durations to be Gamma distributed, with cdf

(5) $$1-\frac{\Gamma (\alpha ,\;t^{⁎}/\lambda )}{\Gamma (\alpha )}$$

where *t*^⁎^ > 0, shape parameter *α* > 0 and scale parameter *λ* > 0 is specified such that *λ* = exp (*x*′*γ*), with parameter *γ* (*k* × 1). The notation *Γ*(∙, ∙) denotes the incomplete gamma function, and *Γ*(∙) the standard gamma function. The duration model nests constant hazards (*α* = 1), as too it is flexible enough to allow for increasing hazards (*α* > 1; i.e. positive duration dependence) and decreasing hazards (*α* < 1; i.e. negative duration dependence). For individuals undertaking *q* events per period, we assume that event duration lengths are mutually independent. Consequently, the aggregate duration *T*^⁎^ is also Gamma distributed, with cdf

(6) $$G(t^{⁎})=1-\frac{\Gamma (\alpha q,\;t^{⁎}/\lambda )}{\Gamma (\alpha q)}$$

note that *E*[*T*^⁎^] = *αqλ* and Var(*T*^⁎^) = *αqλ*^2^. Unlike the Weibull distribution that is more commonly seen in duration analyses, the Gamma distribution is convenient here because it is closed under addition, provided of course that the added components are iid.^5^ Evidence for the suitability of the Gamma assumption was provided earlier in Fig. 1.

The joint cdf of the latent variables (*S*⁎,*T*⁎) is expressed using Sklar's unique representation, namely,

H(s⁎,t⁎)=Pr(S⁎≤s⁎,T∗≤t⁎)=Cθ(F(s⁎),G(t⁎))

where *F* and *G* are the margins specified respectively in (4) and (6). Because it is indexed by a parameter *θ* (in our context this will be a scalar parameter) *C*_*θ*_(∙, ∙) represents a family of copula functions. For example, important here because it emerges as preferred in our empirical application is the family of Frank copulas:

(7) $$C_{\theta }(u,\;\upsilon )=-\theta \log\left(1+\frac{\left(e^{-\theta u}-1\right)\;\left(e^{-\theta \upsilon }-1\right)}{e^{-\theta }-1}\right)\;$$

where *u* and *υ* take values in the unit interval of the real line, and real-valued *θ* is a dependence parameter. For this family of copulas, negative/positive values of *θ* imply a negative/positive association between participation and duration. Independence is nested within the Frank family as the limit case *θ* → 0, for then *C*_*θ*_(*u*, *υ*) → *uv* which is the Product copula.

### 3.4 Likelihood function

For the setting described by the observation rules (2) and (3), along with the copula modelling assumption (7), finds the model to be a member of the Archimedean class of self-selection models studied in Smith (2003). Assuming mutual independence across individuals in our estimation sample, the likelihood function is given by (c.f. Smith (2003, (17)))

(8) $$L=\underset{s=0}{\prod }F\times \underset{s=1}{\prod }\left(1-\frac{\varphi^{\prime }\left(G\right)}{\varphi^{\prime }\left(C_{\theta }\right)}\right)\;g$$

where individual-specific indexes have been dropped throughout merely for convenience. There is a considerable amount of notation behind each term appearing in (8). The notation *Π*_*s* = 0_ forms the product over all non-participants as indicated by *s* = 0, while the product across all participants is formed by *Π*_*s* = 1_. The notation *F* = *F*(0) is, to our specification of the propensity to participate (4), given by

$$F(0)=\Phi \left(-x^{\prime }\beta \right)=1-\Phi \left(x^{\prime }\beta \right)$$

while for durations the notation *G* = *G*(*t*), given in (6), from which *g* = *g*(*t*) is such that

$$g(t)=\frac{\partial }{\partial t}G(t)=\frac{\lambda^{-1}}{\Gamma \left(\alpha q\right)}\exp\left(-\frac{t}{\lambda }\right)\;{\left(\frac{t}{\lambda }\right)}^{\alpha q-1}\text{.}$$

Further notation concerns the copula; namely, *C*_*θ*_ = *C*_*θ*_(*F*, *G*). Finally, let *φ*(∙) be the generator function of copulas of the Archimedean class and *φ*′(∙), that appears in *L*, be its derivative; for details, see Nelsen (2006). In particular, for the Frank family (7),

$$\varphi^{\prime }(r)=\frac{\theta }{1-e^{\theta r}}\text{.}$$

Given the modelling assumptions (4), (6) and (7), *L* is the likelihood function for the parameters *α*, *β*, *γ* and *θ*.

At present the model parameters are identified only because of the non-linearity that is induced in the joint distribution of the observables. Exclusion restrictions amongst the covariates serve to mitigate the problems associated with weak identification such as computational non-convergence and large confidence intervals. In particular, we specify *k*_0_ = 31 covariates in the regression function of the participation model (4), and *k*_1_ = 26 covariates in the regression function of the aggregate duration model (6); neither covariate set nests the other.

Inclusions in the participation regression function relating to individual socioeconomic status include education, income and employment status. Education is assumed to proxy individuals' knowledge relating to the health benefits of actively engaging in sporting activities. The more educated may also be better in producing health (Grossman, 1972). Assuming that participation in sporting activities positively contributes to good health, education may be a contributing factor to health production, lending support to Grossmann's view. Education may also be viewed as a habit formation mechanism whereby individuals who have enjoyed longer periods in the education system may have developed a greater appetite for sports at school when young. For these reasons it is expected that the propensity to participate in sporting activities is increasing in educational attainment. In terms of duration, the effect of education can be interpreted in terms of the opportunity cost of time. The more educated will have a higher opportunity cost of time since their hourly earnings should be higher relative to those with lower education, hence leisure time is more expensive to the highly educated with the effect that those with higher education spend less time in sports conditional on participation; a substitution effect. Assuming sporting activity to be a normal good, then economic theory informs us that as hourly earnings increase, individuals consume more of a normal good; the income effect. The same argument applies for the interpretation of the employment effect on time spent in sporting activity which is controlled for in the duration model. Income is assumed to proxy the ease of financial accessibility to sporting facilities with regards to participation and the effect is expected to be positive. Income and education are both excluded from the duration equation.^6^ Employment status is not only assumed to capture the amount of leisure time at the individual's disposal but more importantly, the individuals' opportunity cost of time. Given that the employed have a higher opportunity cost of time relative to the retired, unemployed and inactive, we would expect to see a negative relationship between the propensity to participate in sports and the employed relative to the retired and unemployed, whilst the effect is unclear relative to the economically inactive who might be inactive due to disability. The effect of employment status on duration is similar to that discussed for education. In order to capture whether poor health is a mitigating factor on sporting participation, a general subjective health variable is included alongside a psychological well being variable, plus an indicator of whether the individual has had an accident in the last 12 months. Participation is expected to decline with deteriorating general health status. This has been evidenced previously by FS and HR, and health reasons have been given as one important reason for a lack of participation (Scottish Executive Education Department, 2006). However, we cannot rule out that individuals in poor health participate in sports as part of a medical recovery process to regain better general health. General health status also features as a determinant of duration, and it is expected that duration increases with increasing general health. Sporting participation has been found to be positively correlated with positive psychological well-being although the direction of causality remains unclear within the exercise psychology literature (Scully et al., 1998). The inclusion of psychological well being in the participation decision is motivated by evidence suggesting that one reason for exercise relate to improved mental health since it offers stress relief and relaxation (Scottish Executive Education Department, 2006). We control for psychological well-being in participation to judge whether it has any significant effect, whilst it is excluded as a factor of duration. The accident indicator is designed to detect if there exist health constraints preventing engagement in sporting activity. Nevertheless, an argument can also be made for the effect to be of the opposite direction given that the particular nature of the injury after an accident may require a medically prescribed exercise regime. A further inclusion for reasons of detecting accessibility constraints is the availability of a car, whilst we assume the availability of a car not to have any effect on duration conditional on participation.

Two area-level indicators are assigned to respondents, where these are constructed by aggregating the data across Scotland's 15 Health Boards: (i) the average BMI, and (ii) the average hours doing sports per week. Both indicators are assumed to pick up peer group influences in relation to diet and exercise. We assume these variables to have a direct effect on participation whilst not having a direct effect on the duration decision. The two area-level indicators can alternatively be thought of as instruments for individual BMI and the time commitment to sporting activity which cannot themselves be included as determinants of participation due to endogeneity problems. The inclusion of the number of children aged 2–15 and the number of children present under the age of two capture childcare and home commitments. The presence of infants is expected to exert a negative effect on participation whilst it is unclear whether the presence of older children inhibits participation or not. We expect the presence of very young children to have a negative effect on duration whilst the effect of older children is unclear. The inclusion of the variable indicating whether the natural mother is still alive serves as a proxy, conditional on having children, for the availability of childcare. We expect the effect on participation to be positive, as well as the effect on duration. The marital status dummies also incorporate an element of family commitment and therefore represent a time constraint to sporting participation. Individuals who are single or divorced, separated or widowed are assumed to be able to manage their leisure time more freely while those who are married face additional family time constraints. We therefore expect married individuals to be less likely to participate relative to singles, and divorced, separated and widowed individuals. The same argument applies to the effect of marital status on duration. In particular for singles we expect relatively more time spent in sporting activity relative to individuals who are married. Given that the separated, divorced and widowed are grouped into a single category, the duration effect remains inconclusive. Participation and duration are assumed to decline with increasing age and men are assumed to have a higher propensity to participate and longer durations of sporting activity relative to women.

Lifestyle factors that impart information about individuals' preferences for health that are thought to impact on participation and duration are captured by a set of variables relating to smoking, drinking and diet status. Smokers are expected to have a lower propensity to participate compared to non- and ex-smokers since they may either not be able to participate due to bad lung function, or because they place lesser value on the healthy benefits derived from sports compared to non- and ex-smokers. Durations should also be negatively related to smoking. The effect of the level of alcohol consumption on participation is, a priori, difficult to gauge. Many sports (especially team sports) have the added benefit of social networking and convey a sense of belonging to an environment that encourages social engagement ‘off the pitch’. In this sense sports may in fact impart an element of fostering risky health behaviour as well. For this reason a positive association between alcohol consumption and participation may be expected. On the other hand, excessive drinking captured here by alcohol consumption over the recommended limit imparts the notion of no preference for health which is associated with a negative effect on participation. Therefore, the direction of the effect is ambiguous a priori. The diet score contains information relating to individual weight as a proxy for health preferences regarding food intake. Individuals with healthier diets and therefore a higher diet score are expected to be more likely to participate relative to those with a less healthy diet score. However, it may also be the case that individuals with a very unhealthy diet score compensate this type of behaviour by a very physically active lifestyle. If this is the case, this should impact positively on duration. On the other hand, if those with unhealthy diet scores are the typical ‘coach potato’ type, the effect on duration should be negative. A final lifestyle variable capturing time use included as a determinant of participation and duration is the number of hours watching television per week. TV watching is sedentary in nature and is believed to have negative effects on both participation and duration.

Inclusions in the duration regression function but excluded from the participation function are the presence of a limiting long-standing illness and non-limiting long-standing illness, with the reference group being no limiting long-standing illness present. Both, limiting and non-limiting long-standing illness may impose a constraint on duration relative to those who do not suffer from either. It may restrict the type of sporting activities the individual may be able to perform and thereby indirectly the duration of the sporting activity. Since the presence of these types of illnesses may not necessarily be a barrier to participation, it may certainly have an effect on duration and we therefore control for the effect in the duration regression function. We further control for vigour in the duration but not the participation regression function. Fig. 2 already evidenced the relationship between vigour and duration and we argue here that it is a vital determinant of duration.

We may have reasons to believe that the strength and the significance of the determinants of participation and duration may differ by gender. An understanding of this is particularly important for policy recommendations. For example, the effect of the number of children present under the age of two may have no direct effect on participation for men but a significantly reducing effect for women. The same argument applies for the effect on duration. If the policy objective is to incentivise women to participate in sports, then this should incorporate the availability of childcare. The model is therefore estimated for men and women separately in addition to a model that takes both men and women into account and captures any gender differences with a gender dummy.

## 4 Results

### 4.1 Parameter estimates

A summary of the maximum likelihood estimation results for the whole sample appear in Table 3. Two sets of estimation results are presented corresponding to: (i) The Independence Model — independence is imposed between *S*⁎ and *T*⁎, and (ii) The Frank model — the association between *S*⁎ and *T*⁎ is described by the Frank family of copulas (7). For each model the estimates are further split across two columns corresponding to the parameters of the participation margin in the first column and the parameters of the duration margin in the second column.^7^

The Frank model nests the Independence model through the restriction *θ* → 0. Testing this restriction rejects the Independence model at any conventional level of significance; for example, the relevant likelihood ratio statistic is *LR* = 286 on a one degree of freedom test. The immediate implication of this result is that participation and duration are associated. The Kendall *τ* statistic (*τ* = *τ*(*θ*))^8^ appearing at the foot of the table indicates a positive association between these variables; the stronger the incentive or propensity to participate in sports activity the longer will be the time spent on activity. These results are also found for the analysis by gender as presented in Table 4 for women and Table 5 for men.

Firstly, consider in isolation the results from the participation component of our preferred Frank model. In regard of age, the distribution of estimates across the age categories (reference group 55–64 years old) behave as intuition would suggest, namely that individuals who actively engage in sports are presented across all age groups, with younger individuals (ages 16–25) more likely to participate. Not surprisingly, the propensity to participate in sports declines with age. There is a significant gender effect that indicates that males on average have a higher propensity to participate relative to females. These findings are consistent with those of HR, DR and FS in their studies. Amongst the lifestyle variables, smokers are significantly less likely to participate in sporting activities relative to non-smokers. This may reflect smokers' lower discount rate for health. On the other hand, ex-smokers are significantly more likely to participate. Anecdotal evidence may argue that giving up smoking is often undertaken in conjunction with a positive change in physical activity behaviour. Interestingly, relative to those who never or occasionally consume alcohol, both groups of drinkers (those that drink over the weekly recommended limit, and those who do not exceed the limit) are more likely to participate. Also, there is no significant difference between these two groups. As such, for individuals who consume alcohol over the limit, this is not a deterrent to engage in sports. This result may support the notion that sports participation serves as a social inclusion or networking device, or that those individuals that consume alcohol are generally social people. Moreover, the argument that those who consume excess amounts of alcohol have no preference for health, at least in relation to sporting activity, is rejected by our data. The positive association between alcohol consumption and sports participation has also been evidenced by FS. The diet and physical activity area measures are both of the expected sign and both are significant determinants of participation. Average BMI in the respondent's Health Board shows a reducing effect on the probability to participate, whilst the average hours spent on sporting activities has a strong positive effect. As such, the results indicate that ‘neighbourhood’ characteristics or peer group effects do have significant implications in terms of sports participation. Hours spent watching television has the expected significant negative effect on participation. We find a significant negative effect of infants on the probability to engage in sports, whilst the number of children aged 2–15 has a significant positive effect. The indicator variable showing whether the natural mother is still alive (a proxy for childcare) is significantly positive in the participation model.

The socioeconomic variables show the following. Higher equivalised household income induces an increased propensity to participate in sports. Low income may therefore act as a barrier to sports participation and any policy aiming to boost numbers of physically active individuals amongst this group needs to take this into account where there are financial barriers to participation (sports club or gym membership and the investment in sporting equipment). Our results show that individuals reporting no educational attainment are less likely to engage in sports relative to their educated counterparts. This lends support to the hypothesis that the more educated have better understanding of the health benefits of sporting activities relative to the uneducated, and supports the use of information initiatives providing awareness of the health benefits of a physically active lifestyle across all groups in society. Secondly, the more educated will have a higher opportunity cost of time assuming that their hourly wages are higher than those of the uneducated. This means that leisure time is relatively more expensive for educated individuals who may therefore wish to substitute away from leisure time activities. Given that the education effect is found to be significantly increasing in education and assuming that sporting activity is a normal good, the results support an income effect rather than a substitution effect. Consistent with our earlier argument on time constraints hampering participation are the results on the economic status indicators, these suggesting that the retired are significantly more likely to participate relative to the employed whereas the effect is insignificant for the inactive and the unemployed. As expected, individuals of very good, good and fair health are more likely to be physically active, with the effect diminishing as the standard of general health declines. Whether an individual has had an accident in the last 12 months is showing a significant positive effect on participation suggesting that sporting activity may be gainfully used for the purposes of rehabilitation.

The analysis by gender reveals some further insights into sporting participation. The magnitude of the effect of household income is slightly higher for men. Whereas marital status has no effect on sports participation for men, it is highly significant and positive for singles and the group of divorced, widowed or separated women relative to married women, suggesting that home production is a barrier to sports participation for married women. Related to this is the observation that the number of infants is a highly significant deterrent for women to participate in sports but not the number of children aged 2–15. For men the number of children of any age is not a contributing factor inhibiting sports participation. This suggests firstly that policies directed to incentivise women with infants to participate in sports needs to address childcare issues. Conditional on the presence of infants, the proxy for childcare (natural mother alive) increases participation in sports for women, but the effect is insignificant for men. Hours watching TV per week is clearly a barrier to sports participation for both men and women, but decidedly more so for women. The impact of education for men and women separately is similar to that found for the sample as a whole. As such there is no gender difference in the propensity to engage in sports across genders for the educated. However, uneducated men and women are less likely to participate compared to more educated men and women. There are significant differences between men and women relating to the impact of employment status. For men there are no significant differences across employed, unemployed, retired and inactive, but amongst women those that are retired and unemployed are more likely to participate relative to employed women. The unemployed and retired in general have more leisure time at their disposal compared to the employed, so they are expected to participate more in leisure activities such as sports due to lower opportunity costs. The insignificant effect for men suggests that there is scope to introduce policies tailored to incentivise retired and unemployed men to participate in sports. The impact of lifestyles also impacts differently on men and women. Smoking status is not significant for men whilst smoking is a highly significant barrier to sports participation for women relative to non-smoking women. Additionally, women who gave up smoking in the past are more likely to engage in sports compared to non-smoking women. For women, the consumption of alcohol has a positive effect on participation whether it is under or over the recommended limit compared to women who never or occasionally drink. The regular drinking of alcohol over the limit does not impact significantly on sports participation for men although the propensity to participate is higher for men drinking under the recommended limit relative to men who never or occasionally drink. The results on alcohol consumption seem to suggest that alcohol consumption is not a barrier to sports participation and that individuals who do not, or only occasionally drink (the healthy ones in terms of this type of lifestyle), are the ones that have a lower propensity to participate in sports. A healthy diet score significantly affects sports participation positively for both men and women. As seen for the sample as a whole, sports participation is increasing in general health. Men of very good, good and fair general health have a higher propensity to participate in sports than men of bad health. For women the same holds true although only the ‘very good’ and ‘good’ general health dummies show a significant effect. Psychological well-being is not a significant determinant of sports participation for men whilst women of fair psychological well-being are more likely to participate relative to women of bad psychological well-being. Finally, the analysis by gender shows that peer group effects are important for men and women in relation to the average hours of sports recorded in the health board the respondent lives in. As these increase so does the likelihood of participation in sports. Interestingly the average BMI in the health board only has a significantly strong reducing effect on participation for women, not for men for whom this effect is found to be insignificant. In general this shows that a physically active ‘neighbourhood’ has beneficial effects on individuals belonging to such a ‘neighbourhood’. However, a fat ‘neighbourhood’ in terms of BMI is particularly harmful to women's likelihood of sports uptake implying that policies should focus on promoting female sports in areas of high overweight and obesity prevalence.

Next, consider the duration models in isolation of the participation model. Our results for the whole sample show that on average men spend significantly more time undertaking sports activities than women. The age effect is such that duration increases and is highest amongst the 25–34 year olds, and thereafter duration times decline with increasing age. However, the analysis by gender reveals that there are no significant age effects for men whereas women of age groups younger than the 55–64 year olds have significantly longer duration. Durations are also highest for individuals who are single, although it is evident from Tables 4 and 5 that the effect is only significant for women. For men it is found that the divorced, widowed or separated have significantly lower durations relative to married men. For the whole sample, it is the number of infants under the age of two that impacts negatively on duration. While this had no bearing on the propensity to participate for men, in terms of the effect on duration we find a significant negative effect of infants on duration for both genders. Whilst it is the case that infants are not a barrier to participation for men, the time available for sports activities is significantly reduced for both men and women. While we can uncover no effect of our childcare availability proxy ‘natural mother alive’ on duration for the whole sample, the results by gender suggest a significantly positive effect on duration for men but no significant effect for women. As the number of hours watching television increases duration decreases as expected. This holds true for both men and women. Whilst employment status significantly impacts on participation, it has no significant effect on duration. In terms of self-reported general health there are significant positive effects for those of very good, good and fair health relative to those of bad general health, with durations being largest for individuals (irrespective of gender) with very good self-reported health. Individuals subject to a long-standing limiting illness have significantly reduced duration spells on average. Being a smoker significantly reduces duration, opposite to that of the ex-smoker who has a significantly increased duration spell, both effects are relative to non-smokers. The decision to give up smoking often involves taking up a sport as a potentially psychological incentive to avoid reverting back to smoking, and the witnessed duration effect may be an indication of a need to drive harder. However, the analysis by gender reveals this effect only to be significant for women. On the other hand, alcohol consumption has no significant effect on duration time for the whole sample. Looking at the results for men and women in isolation, the result remains unchanged for men whereas for women regular alcohol consumption over the recommended limit has a significant positive effect on duration. Given that high alcohol consumption is no significant deterrent to sports participation, and conditional on participation duration is significantly higher relative to those who never or occasionally drink alcohol, it is unclear whether this effect results from a strategy compensating unhealthy with healthy behaviour by women. A healthy diet score whilst having a positive impact on participation has no significant impact on duration. Finally, compared to those who undertake sports with low levels of vigour, those with moderate or high vigour have significantly lower durations. The result holds irrespective of gender. This is what we would expect since moderate and vigorous activity also means that one ‘burns out’ after a shorter period of time. Descriptive evidence on this relationship has already been presented in Fig. 2.

### 4.2 Conditional analyses

Scottish government guidelines recommend that adults undertake a half hour of moderate vigour physical activity on at least 5 days per week in order to maintain a healthy weight. We can assess aspects of the Scottish guideline by using predictions computed from our model of sporting activities as inputs into an associated health condition model. As our illustration, we examine the implications on durations resulting from shifting participants from low to higher degrees of vigour associated with their sporting activities. We then use our predictions as inputs into a weight health outcome model.^9^

There are two parts to this analysis the first of which involves the model we have already developed. In particular, we assess duration in terms of the conditional mean *E*[*T*^⁎^|*S*^⁎^ > 0], i.e. the conditional expectation of aggregate duration given occurrence of participation in sporting activities. In the absence of any sample selection effect conditional and marginal analyses coincide, because if *T*⁎ and *S*⁎ are independent then

(9) $$E[T^{⁎}|S^{⁎}>0]=E[T^{⁎}]=\alpha q\lambda \text{.}$$

The evidence from our data does not however support the case for independence. For our preferred Frank model, the following incomplete integral expresses the conditional expectation:

(10) $$E[T^{⁎}|S^{⁎}>0]=\Phi {\left(x^{\prime }\beta \right)}^{-1}\left(\alpha q\lambda -\int_{0}^{\infty }t\frac{1-\exp(\theta C_{\theta })}{1-\exp(\theta G)}gdt\right)\;$$

where the notation is the same as was used in presenting *L*. Numerical methods are required when evaluating (10).

The next component we require is a weight model for which we use a standard linear regression model with body mass index (BMI) as our dependent variable; the fitted model is presented in Table 6. The first feature worth noting is that undertaking sport (participation = 1) significantly reduces BMI relative to non-participants. The next feature concerns the three interactions between aggregate duration (measured in hours per week) and vigour. The interaction associated with moderate vigour does not have any significant effect on BMI, implying that BMI is maintained irrespective of the time spent on sport; the other two interactions do however have significant, but opposite-signed effects. The duration/low vigour interaction on average significantly increases BMI, thereby lending support to the Scottish guidelines that aim to have individuals attempt more vigorous activity. The duration/high vigour interaction enters such that on average there is a significant decline in BMI for each hour spent playing sport, further accentuating the health benefit due to participation.

Our first example concerns a male, aged between 35 and 44, married with no children and in very good health.^10^ Working in units of hours per week and fixing their participation at just the one event per week (*q* = 1) our preferred Frank model predicts aggregate duration in each case as:

$$\begin{array}{l} \hat{E}[T^{⁎}|S^{⁎}>0,\;q=1,\;\text{ low vigour}]=2.5\text{ h/wk}\\ \hat{E}[T^{⁎}|S^{⁎}>0,\;q=1,\;\text{ moderate vigour}]=1.0\text{ h/wk}\\ \hat{E}[T^{⁎}|S^{⁎}>0,\;q=1,\;\text{ high vigour}]=1.2\text{ h/wk} \end{array}$$

We see that, amongst participants, inducing an increase in vigour from the lowest level results in a large decline in duration, and one that clearly brings this representative individual below the guideline duration of 2.5 h per week. To compensate in terms of multiplicity of events, consider the result

$$\hat{E}[T^{⁎}|S^{⁎}>0,\;q=2.5,\;\text{ moderate vigour}]=2.4\text{ h/wk}\text{.}$$

Here we see that to maintain the guideline duration, individuals need to be encouraged to bolster the number of events they undertake. However, it is worth recalling that our BMI model determines that duration is irrelevant in maintaining the level of BMI for sport of moderate vigour.

Our second example concerns a female, aged between 35 and 44, married with 3 children (one under two), and in good health.^11^ Our preferred Frank model predicts in her case:

$$\hat{E}[T^{⁎}|S^{⁎}>0,\;q=1,\;\text{ low vigour}]=1.5\text{ h/wk}\text{.}$$

A similar pattern emerges to before with large declines in duration when vigour is increased from a low degree of effort; for example,

$$\hat{E}[T^{⁎}|S^{⁎}>0,\;q=1,\;\text{ moderate vigour}]=0.6\text{ h/wk}\text{.}$$

Required now would be a five-fold increase in the number of events if the recommended guidelines were to be achieved. Both this and the previous example show that there are large trade-offs between duration and increases in vigour.

Our third example concerns a male, aged between 16 and 25, single, in very good health, but a smoker and regular over limit drinker.^12^ Our preferred Frank model predicts in his case

$$\begin{array}{l} \hat{E}[T^{⁎}|S^{⁎}>0,\;q=5,\;\text{ high vigour, smoker}]=6.7\text{ h/wk}\\ \hat{E}[T^{⁎}|S^{⁎}>0,\;q=5,\;\text{ high vigour, ex-smoker}]=7.7\text{ h/wk}\text{.} \end{array}$$

An intervention bringing about a shift from smoker to ex-smoker results on average in an increase in duration, which in the case illustrated amounts to one further hour per week. In terms of BMI outcome, the additional time devoted to sporting activities serves to decrease BMI, but the change in status to ex-smoker offsets to increase BMI, holding all else constant. Given the characteristics assigned to the individual, the BMI regression predicts 24.28 on average when the individual is a smoker, and 25.34 when smoking status changes to non-smoker; an overall increase of just over one BMI point, but one that manages to shift the individual out of the healthy weight bracket up into the next overweight category. Arguably, the increased risks of obesity-related diseases as a result of the slight shift in predicted BMI will be more than compensated by the across-the-board reduction in health risks resulting from quitting smoking.

## 5 Conclusion

In this paper we examined the link between participation in physical activity and time spent. The motivation to do so derived from the premise that these two components cannot be studied independently of each other. We therefore opted to model the relationship via a sample selection model, using flexible parametric forms based on copulas. Our modelling results provide compelling and significant evidence in favour of there not only being a link between the components, but that the direction of the association is positive.

Our model results support findings on sports participation and duration given in the previous literature. Sports participation significantly reduces with increasing age, and men are more likely to participate in sports relative to women. Household characteristics such as the presence of infants are found to impact negatively on sports participation in general, and married individuals are less likely to participate relative to non-married individuals. The analysis by gender further reveals that the effect of infants and marital status is only significant for women suggesting that physical activity health improvement programmes should take this into account by offering, for example, childcare, given that conditional on the presence of infants, our proxy for childcare has a significantly positive effect on participation for women. Low income is revealed to be a significant barrier to sports participation in the study sample. This holds true for men and for women. Policies directed at inducing sports participation should therefore aim to reduce the financial inaccessibility of sports to low income earners. Our results further suggest that those who are more educated have a higher propensity to participate in sports and that this results holds for both men and women. We argued that this may be due to the more educated being more aware of the beneficial health effects of sports participation. Informational policy campaigns relating to the improved health gains from sports across society may therefore be an effective way of reaching out to those of lower education. The employed are significantly less likely to participate in sports relative to the retired. This lends support to the hypothesis that time constraints are a significant deterrent to sports participation. Overall, these results show that the economic factors are important determinants of sports participation for men and women, where it is also found that income positively affects duration for both genders. As expected, lifestyles also impact on sports participation and duration. Smoking has the anticipated negative effect on both, while higher levels of alcohol consumption have an increasing effect. This refutes the belief that individuals who consume high levels of alcohol have no preference for health. This result may be interpreted in terms of sports encouraging social networks, especially team sports, or that an unhealthy behaviour may be compensated for by vigorously pursuing a healthy behaviour. The ‘neighbourhood’ or peer group effects reveal that an active and healthy ‘neighbourhood’ in terms of BMI has positive participation and duration effects. However, a ‘fat’ peer group is particularly harmful for sports participation for women. Policies should therefore focus on promoting female sports and sports inclusion in areas of high overweight and obesity prevalence.

Our model has limitations as well. We have elected to focus on physical sporting activities during leisure time and excluded physical activities in the home, in market production, and in the everyday living activities. As such, our modelling results excludes individuals who do not participate in the sports our analysis is based on, which may cause us to underestimate the health effects that would prevail had our model accounted for all sources of physical activity. Further, we did not disaggregate our analysis by types of sports undertaken. Future work might take partitionings like this into account since they will aid in identifying the differing health effects due to different types of sports.

## Figures and Tables

**Table 1:** Vigour by sporting events.

	Events						Total
	1–4	5–8	9–12	13–16	17–20	> 20	
Light	153	52	32	14	15	21	287
Moderate	334	116	47	40	14	40	591
High	544	307	198	124	79	197	1,449
Total	1,031	475	277	178	108	258	2,327

**Table 2:** Descriptive statistics.

	Participants		Non-participants		Section 4.2 simulations		
	Mean	Std dev	Mean	Std dev	#1	#2	#3
Gender (male = 1)	0.486		0.433		1	0	1
Age 16 to 24	0.142		0.064		0	0	1
Age 25 to 34	0.211		0.134		0		
Age 35 to 44	0.290		0.234		1	1	0
Age 45 to 54	0.199		0.269		0		
Age 55 to 64 ⁎	0.157		0.298		0		
Ln equivalised household income	10.010	0.822	9.719	0.810	10	10	9
Married/cohabiting ⁎	0.654		0.710		1	1	0
Single	0.242		0.150		0	0	1
Divorced/separated/widowed	0.104		0.141		0		
No. children aged 2–15	0.630	0.931	0.469	0.856	0	2	0
No. children under age 2	0.067	0.256	0.070	0.273	0	1	0
Natural mother alive	0.727		0.546		1		
Hours watching TV per week	5.822	3.225	7.315	4.704	5.822		
Car available in household	0.847		0.761		1	1	0
Education ⁎	0.852		0.641		1	1	0
No education	0.148		0.386		0	0	1
Employed ⁎	0.749		0.622		1	0	0
Retired	0.049		0.072		0		
Unemployed	0.062		0.047		0	0	1
Economically inactive	0.141		0.260		0	1	0
General health: Very good	0.449		0.286		1	0	1
Good	0.413		0.395		0	1	0
Fair	0.113		0.216		0		
Bad ⁎	0.025		0.104		0		
Psychological wellbeing: Good	0.643		0.605		1	0	1
Fair	0.212		0.173		0	1	0
Bad ⁎	0.119		0.180		0		
Missing	0.026		0.042		0		
Longstanding illness: Limiting	0.161		0.302		0		
Non-limiting	0.146		0.147		0		
None ⁎	0.693		0.552		1		
Accident	0.130		0.106		0		
Health Board average weekly hours sports activity	1.870	0.218	1.815	0.276	1.870		
Health Board average BMI	27.032	0.255	27.095	0.309	27.032		
Never smoked ⁎	0.507		0.388		1	1	0
Smoker	0.226		0.355		0	0	1
Ex-smoker	0.255		0.233		0		
Occasional/never drinker ⁎	0.291		0.409		0	1	0
Regular under limit drinker	0.454		0.387		1	0	0
Regular over limit drinker	0.242		0.197		0	0	1
Healthy diet score	18.450	4.856	17.473	4.873	18	18	14
Total hours doing sports per week	2.488	3.400					
Low vigour ⁎	0.123						
Moderate vigour	0.254						
High vigour	0.623						
Sample size	2327		2053				

**Table 3:** Maximum likelihood estimates.

	Independence model		Frank model	
	Participation	Duration	Participation	Duration
Constant	3.338	1.493⁎⁎	3.277	1.325⁎⁎
Gender (male = 1)	0.190⁎⁎	0.119⁎⁎	0.200⁎⁎	0.172⁎⁎
Age 16 to 24	0.771⁎⁎	0.024	0.697⁎⁎	0.203⁎⁎
Age 25 to 34	0.542⁎⁎	0.057	0.491⁎⁎	0.187⁎⁎
Age 35 to 44	0.381⁎⁎	0.044	0.352⁎⁎	0.134⁎
Age 45 to 54	0.090	0.083	0.096	0.133⁎⁎
Ln equivalised household income	0.110⁎⁎		0.107⁎⁎	
Single	0.222⁎⁎	0.124⁎⁎	0.238⁎⁎	0.175⁎⁎
Divorced/separated/widowed	0.158⁎	− 0.154⁎⁎	0.122⁎	− 0.116⁎⁎
No. children aged 2–15	0.079⁎⁎	− 0.039⁎	0.042	− 0.029
No. children under age 2	− 0.181⁎⁎	− 0.226⁎⁎	− 0.223⁎⁎	− 0.285⁎⁎
Natural mother alive	0.106⁎	− 0.005	0.102⁎	0.038
Hours watching TV per week	− 0.036⁎⁎	− 0.008⁎	− 0.037⁎⁎	− 0.019⁎⁎
Car available in household	0.095		0.015	
No education	− 0.337⁎⁎		− 0.325⁎⁎	
Retired	0.289⁎⁎	− 0.076	0.234⁎	− 0.024
Unemployed	0.124	0.050	0.185⁎	0.073
Economically inactive	− 0.056	0.003	− 0.050	− 0.040
General health: Very good	0.613⁎⁎	0.381⁎⁎	0.608⁎⁎	0.550⁎⁎
Good	0.437⁎⁎	0.328⁎⁎	0.459⁎⁎	0.433⁎⁎
Fair	0.233⁎	0.317⁎⁎	0.240⁎	0.359⁎⁎
Psychological wellbeing: Good	0.022		− 0.009	
Fair	0.192⁎⁎		0.164⁎⁎	
Missing	− 0.236		− 0.252	
Longstanding illness: Limiting		− 0.088⁎		− 0.098⁎⁎
Non-limiting		0.019		0.020
Accident	0.130⁎		0.058⁎	
Health Board average hours phys activity	0.412⁎⁎		0.309⁎⁎	
Health Board average BMI	− 0.241⁎⁎		− 0.226⁎⁎	
Smoker	− 0.194⁎⁎	− 0.029	− 0.155⁎⁎	− 0.076⁎
Ex-smoker	0.101⁎	0.044	0.094⁎	0.066⁎
Regular under limit drinker	0.172⁎⁎	0.005	0.142⁎⁎	0.034
Regular over limit drinker	0.183⁎⁎	0.018	0.150⁎⁎	0.046
Healthy diet score	0.029⁎⁎	− 0.004	0.028⁎⁎	0.002
Moderate vigour		− 0.780⁎⁎		− 0.861⁎⁎
High vigour		− 0.675⁎⁎		− 0.710⁎⁎
Gamma shape *α*		0.315⁎⁎		0.218⁎⁎
Copula theta *θ*				5.597⁎⁎
Log*L*	− 9298.29		− 9155.25	
Kendall tau *τ*				0.492⁎⁎

Notes: Significance from zero at the 5% level is indicated by ⁎, and at the 1% level by ⁎⁎. Units of measure: hours over a 4 week period. Sample size *n* = 4380, number of participants *n*_1_ = 2327.

**Table 4:** Maximum likelihood estimates: women.

	Independence model		Frank model	
	Participation	Duration	Participation	Duration
Constant	5.793	0.802⁎⁎	5.237	0.697⁎⁎
Age 16 to 24	0.770⁎⁎	0.099	0.698⁎⁎	0.244⁎
Age 25 to 34	0.574⁎⁎	0.102	0.579⁎⁎	0.234⁎⁎
Age 35 to 44	0.457⁎⁎	0.047	0.446⁎⁎	0.137
Age 45 to 54	0.124	0.159⁎	0.193⁎	0.210⁎⁎
Ln equivalised household income	0.090⁎		0.095⁎	
Single	0.302⁎⁎	0.182⁎⁎	0.330⁎⁎	0.234⁎⁎
Divorced/separated/widowed	0.266⁎⁎	− 0.102⁎	0.229⁎⁎	− 0.081
No. children aged 2–15	0.029	− 0.042⁎	− 0.004	− 0.041
No. children under age 2	− 0.312⁎⁎	− 0.305⁎⁎	− 0.337⁎⁎	− 0.416⁎⁎
Natural mother alive	0.179⁎⁎	− 0.114⁎	0.158⁎	− 0.046
Hours watching TV per week	− 0.043⁎⁎	− 0.008	− 0.044⁎⁎	− 0.022⁎⁎
Car available in household	0.105		0.044	
No education	− 0.341⁎⁎		− 0.306⁎⁎	
Retired	0.297⁎	0.065	0.266⁎	0.093
Unemployed	0.215	0.047	0.303⁎	0.108
Economically inactive	0.001	− 0.006	− 0.009	− 0.022
General health: Very good	0.425⁎⁎	0.478⁎⁎	0.410⁎⁎	0.566⁎⁎
Good	0.299⁎	0.371⁎⁎	0.318⁎⁎	0.420⁎⁎
Fair	0.178	0.383⁎⁎	0.183	0.387⁎⁎
Psychological wellbeing: Good	0.103		0.037	
Fair	0.168		0.167⁎	
Missing	− 0.181		− 0.231	
Limiting longstanding illness		− 0.034		− 0.058
Non-limiting longstanding illness		0.148⁎⁎		0.148⁎⁎
Accident	0.108		0.102⁎⁎	
Health Board average hours phys activity	0.456⁎⁎		0.330⁎⁎	
Health Board average BMI	− 0.333⁎⁎		− 0.301⁎⁎	
Smoker	− 0.254⁎⁎	− 0.067	− 0.211⁎⁎	− 0.120⁎⁎
Ex-smoker	0.125	0.136⁎⁎	0.139⁎	0.141⁎⁎
Regular under limit drinker	0.176⁎⁎	0.023	0.149⁎	0.043
Regular over limit drinker	0.310⁎⁎	0.091⁎	0.295⁎⁎	0.121⁎
Healthy diet score	0.038⁎⁎	− 0.008⁎	0.036⁎⁎	0.000
Moderate vigour		− 0.145⁎		− 0.205⁎⁎
High vigour		− 0.157⁎⁎		− 0.143⁎
Gamma shape *α*				0.239⁎⁎
Copula theta *θ*				6.067⁎⁎
Log*L*	− 4549.57		− 4488.07	
Kendall tau *τ*				0.518⁎⁎

Notes: Significance from zero at the 5% level is indicated by ⁎, and at the 1% level by ⁎⁎. Units of measure: hours over a 4 week period. Sample size *n* = 2360, number of participants *n*_1_ = 1196.

**Table 5:** Maximum likelihood estimates: men.

	Independence model		Frank model	
	Participation	Duration	Participation	Duration
Constant	0.444	1.984⁎⁎	1.065	1.774⁎⁎
Age 16 to 24	0.825⁎⁎	− 0.021	0.784⁎⁎	0.195
Age 25 to 34	0.567⁎⁎	0.026	0.482⁎⁎	0.157
Age 35 to 44	0.324⁎⁎	0.030	0.289⁎⁎	0.103
Age 45 to 54	0.062	0.030	0.021	0.057
Ln equivalised household income	0.136⁎⁎		0.108⁎⁎	
Single	0.137	0.050	0.126	0.102
Divorced/separated/widowed	− 0.049	− 0.197⁎	− 0.086	− 0.164⁎
No. children aged 2–15	0.123⁎⁎	− 0.020	0.064	0.001
No. children under age 2	− 0.013	− 0.149⁎	− 0.094	− 0.174⁎
Natural mother alive	− 0.006	0.106⁎	0.005	0.141⁎⁎
Hours watching TV per week	− 0.029⁎⁎	− 0.010	− 0.029⁎⁎	− 0.019⁎⁎
Car available in household	0.056		− 0.023	
No education	− 0.335⁎⁎		− 0.335⁎⁎	
Retired	0.280	− 0.099	0.223	− 0.031
Unemployed	0.017	0.071	0.053	0.053
Economically inactive	− 0.067	0.101	− 0.041	0.032
General health: Very good	0.837⁎⁎	0.326⁎	0.892⁎⁎	0.600⁎⁎
Good	0.610⁎⁎	0.304⁎	0.669⁎⁎	0.487⁎⁎
Fair	0.294	0.219	0.344⁎	0.324⁎⁎
Psychological wellbeing:				
Good	− 0.067		− 0.088	
Fair	0.220⁎		0.122	
Missing	− 0.333		− 0.313	
Limiting longstanding illness		− 0.093		− 0.090
Non-limiting longstanding illness		− 0.095		− 0.091
Accident	0.132		0.016	
Health Board average hours phys activity	0.368⁎⁎		0.273⁎⁎	
Health Board average BMI	− 0.128		− 0.132	
Smoker	− 0.124	0.015	− 0.092	− 0.034
Ex-smoker	0.090	0.020	0.076	0.060
Regular under limit drinker	0.167⁎	− 0.047	0.134⁎	0.003
Regular over limit drinker	0.084	− 0.078	0.023	− 0.044
Healthy diet score	0.019⁎⁎	0.000	0.020⁎⁎	0.005
Moderate vigour		− 1.308⁎		− 1.388⁎⁎
High vigour		− 1.029⁎		− 1.074⁎⁎
Gamma shape *α*		0.303		0.210⁎⁎
Copula theta *θ*				6.156⁎⁎
Log*L*	− 4627.95		− 4552.96	
Kendall tau *τ*				0.522⁎⁎

Notes: Significance from zero at the 5% level is indicated by ⁎, and at the 1% level by ⁎⁎. Units of measure: hours over a 4 week period. Sample size *n* = 2020, number of participants *n*_1_ = 1131.

**Table 6:** BMI regression.

	Coefficient estimate	Std. error
Constant	29.682	(0.803)⁎⁎⁎
Gender (male = 1)	0.364	(0.127)⁎⁎⁎
Age 16–24	− 2.930	(0.257)⁎⁎⁎
Age 25–34	− 1.625	(0.212)⁎⁎⁎
Age 35–44	− 1.017	(0.184)⁎⁎⁎
Age 45–54	− 0.575	(0.183)⁎⁎⁎
Ln equivalised household income	− 0.058	(0.078)
Ex-smoker	0.313	(0.156)⁎⁎
Smoker	− 0.829	(0.154)⁎⁎⁎
Regular over limit drinker	− 0.134	(0.172)
Regular under limit drinker	− 0.664	(0.149)⁎⁎⁎
Healthy diet score	− 0.024	(0.014)⁎
Participation	− 0.516	(0.144)⁎⁎⁎
Duration in hours/week × low vigour	0.101	(0.035)⁎⁎⁎
Duration in hours/week × moderate vigour	− 0.035	(0.055)
Duration in hours/week × high vigour	− 0.075	(0.025)⁎⁎⁎
Observations	4380	
*R*^2^	0.07	

Notes: Significance from zero at the 10% level is indicated by ⁎, at the 5% level by ⁎⁎, and at the 1% level by ⁎⁎⁎.

## Footnotes

1 Ideally, we would prefer less aggregated diary data, i.e. duration *T* and vigour *V* recorded on each event, for then a time use panel dataset could be constructed, however, that level of detail is not available within the SHeS.

2 For any one event to be included into calculation the stipulation set down in interview was that the activity had to be undertaken for at least 15 min on any given day.

3 The most frequently reported sports were swimming, cycling, workout/gym/exercise bike/weight training, aerobics/keep fit/gymnastics/dance for fitness, any other type of dancing, running/jogging, football/rugby, badminton/tennis, squash, and exercises (e.g. press-ups and sit-ups). The entire list of reported sports contained a further 98 types in total.

4 Area level indicators have been used previously as instrumental variables. For example, Morris (2007) used area level indicators to instrument for obesity, asserting that they show peer group effects.

5 Care needs to be exercised when varying the period in which the duration *t*⁎ is measured, because any change also scales the per period event count *q*. As the shape parameter *α* is unitless and *αq* in (6) is invariant, the apparent effect is to introduce the inverse scaling into *α* Note also that varying the measure of *t*⁎ in terms of time unit and/or period affects only the intercept in *x*′*γ*. This is because the model's scale parameter is specified as *λ* = exp(*x*′*γ*).

6 We have tested for the effect of income and education in the duration regression but uncovered no significant effects on duration, even though theory may tell us otherwise.

7 Other models were estimated and their results are available upon request. However, relative to the Frank model they were worse-fitting. The Frank model is our preferred outcome while the Independence model represents our baseline.

8 For Frank's copula *τ* = *τ*(*θ*) = 1 + 4(*D*(*θ*) − 1)/*θ* where the Debye function *D*(*θ*) = *θ*^− 1^∫_0_^*θ*^*t*(*e*^*t*^ − 1)^− 1^*dt* is easily numerically computed. Note the symmetry *τ*(− *θ*) = *τ*(*θ*), as well as the limiting cases: *τ* → ± 1 as *θ* → ± ∞ and *τ* → 0 as *θ* → 0.

9 Babraj et al. (2009) provides one example of the usefulness of relating sports participation and duration to a health condition. They investigated the effect of short duration–high intensity exercise training on insulin action and glycemic control effects in young sedentary men aged 19–23. They found that physical exercise undertaken with low duration brings about a positive health effect when coupled with high intensity. This is encouraging for individuals who may be constrained to follow a time intensive exercise programme.

10 The full list of attributes that are assigned appears under the heading “#1” in the last column of Table 2.

11 The full list of attributes that are assigned appears under the heading “#2” in the last column of Table 2.

12 The full list of attributes that are assigned appears under the heading “#3” in the last column of Table 2.

## References

[bib1] Ainsworth B.E., Haskell W.L., Whitt M.C., Melicia C., Irwin M.L., Swarts A.M., Strath S.J., O'Brien W.L., Basset D.R., Schmitz K.H., Emplaincourt P.O., Jacobs D.R., Leon A.S. (2000). Compendium of physical activities: an update of activity codes and MET intensities. Journal of Medical Science and Sports Exercise.

[bib2] Babraj J.A., Vollaard N.B.J., Keast C., Guppy F.M., Cottrell G., Timmons J.A. (2009). Extremely short duration high intensity training substantially improves insulin action in young sedentary males. BMC Endocrine Disorders.

[bib3] Becker G.S. (1965). A theory of the allocation of time. The Economic Journal.

[bib4] Brownson R.C., Baker E.A., Houseman R.A., Brennan L.K., Bacak S.J. (2001). Environmental and policy determinants of physical activity in the United States. American Journal of Public Health.

[bib5] Brownson R.C., Boehmer T.K., Luke D.A. (2005). Declining rates of physical activity in the United States: what are the contributors?. Annual Review of Public Health.

[bib6] Caspersen C.J., Powell K.E., Christenson G.M. (1985). Physical activity, exercise, and physical fitness: definitions and distinctions for health-related research. Public Health Report.

[bib7] Cawley J. (2004). An economic framework for understanding physical activity and eating behaviours. American Journal of Preventive Medicine.

[bib8] Downward P., Riordan J. (2007). Social interactions and the demand for sport: an economic analysis. Contemporary Economic Policy.

[bib9] Ewing R., Schmid T., Killingsworth R., Zlot A., Raudenbush S. (2003). Relationship between urban sprawl and physical activity, obesity, and morbidity. The Science of Health Promotion.

[bib10] Farrell L., Shields M. (2002). Investigating the economic and demographic determinants of sporting participation in England. Journal of the Royal Statistical Society: Series A.

[bib11] French S.A., Story M., Jeffery R.W. (2001). Environmental influences on eating and physical activity. Annual Review of Public Health.

[bib12] Gronau R. (1974). Wage comparisons — a selectivity bias. Journal of Political Economy.

[bib13] Grossman M. (1972). On the concept of health capital and the demand for health. Journal of Political Economy.

[bib14] Heckman J. (1974). Shadow prices, market wages, and labour supply. Econometrica.

[bib15] Hill J.O., Wyatt H.R., Reed G.W., Peters J.C. (2003). Obesity and the environment: where do we go from here?. Science.

[bib16] Hillsdon M., Foster C., Cavill N., Crombie H., Naidoo B. (2004). The effectiveness of interventions of increasing physical activity amongst adults: a review of reviews. http://www.nice.org.uk/niceMedia/pdf/physical_activity_adults_eb.pdf.

[bib17] Hu F.B., Hu F.B. (2008). Physical activity measurements. Obesity.

[bib18] Humphreys B.R., Ruseski J.E. (2006). Economic determinants of participation in physical activity and sports. International Association of Sports Economists Working Paper Series, 06-13.

[bib19] Lakdawalla D., Philipson T., Bhattacharya J. (2005). Welfare-enhancing technological change and the growth of obesity. The American Economic Review.

[bib20] Morris S. (2007). The impact of obesity on employment. Labour Economics.

[bib21] Nelsen R.B. (2006). An introduction to copulas.

[bib22] Philipson T., Posner R.A. (2004). The long run growth in obesity as a function of technological change. Perspectives in Biology and Medicine.

[bib23] Scottish Executive (2003). *Improving Health in Scotland: The Challenge*. Edinburgh. [Available from http://www.scotland.gov.uk/Resource/Doc/47034/0013854.pdf].

[bib24] Scottish Executive Education Department (2006). *Sport, exercise and physical activity: Public participation, barriers and attitudes*. [Available from http://www.scotland.gov.uk/Resource/Doc/932/0041468.pdf].

[bib25] Scottish Office Department of Health (1997). *Scottish Health Survey 1995*. Volume 1: Findings & Volume II: Technical Report. Edited by Dong, W. and Erens, B. Edinburgh. [Available from http://www.sehd.scot.nhs.uk/publications/sh5/sh5-00.htm].

[bib26] Scully D., Kremer J., Meade M.M., Graham R., Dudgeon K. (1998). Physical exercise and psychological well being: a critical review. British Journal of Sports Medicine.

[bib27] Sklar A. (1959). Fonctions de répartition à *n* dimensions et leurs marges. Publications de l'Institute de Statistique de l'Universite de Paris.

[bib28] Sklar A. (1973). Random variables, joint distributions, and copulas. Kybernetika.

[bib29] Smith M.D. (2003). Modelling sample selection using Archimedean copulas. The Econometrics Journal.

[bib30] Sturm R. (2004). The economics of physical activity. American Journal of Preventive Medicine.

[bib31] Welk G.J. (2005). Physical Activity Assessments for Health-Related Research.

[bib32] World Health Organisation Europe (2007). *The Challenge of Obesity in the WHO European Region and the Strategies for Response*. [Available from http://www.euro.who.int/document/E90711.pdf].

[bib33] World Health Organisation (2002). *The World Health Report 2002. Reducing Risks, Promoting Healthy Lifes*. [Available from http://www.who.int/whr/2002/en/whr02_en.pdf]. 10.1080/1357628031000116808 14741909

[bib34] World Health Organisation (2005). *Preventing Chronic Disease: A Vital Investment*. [Available from http://www.who.int/chp/chronic_disease_report/en/].

